# 21色流式检测人非小细胞肺癌组织中免疫细胞亚群方案的建立

**DOI:** 10.3779/j.issn.1009-3419.2024.102.02

**Published:** 2024-01-20

**Authors:** Tingting GUO, Hongguan XIE

**Affiliations:** ^1^610059 成都，成都理工大学生态环境学院（郭婷婷，谢鸿观）; ^1^College of Ecology and Environment, Chengdu University of Technology, Chengdu 610059, China; ^2^610041 成都，四川大学华西医院呼吸健康研究所（郭婷婷）; ^2^Institute of Respiratory Health, West China Hospital, Sichuan University, Chengdu 610041, China

**Keywords:** 肺肿瘤, 外周血单个核细胞, 免疫表型, 多色流式, Lung neoplasms, Peripheral blood mononuclear cells, Immunophenotype, Multicolor flow cytometry

## Abstract

**背景与目的** 肺癌组织的免疫微环境已成为关注的重点，随着多色流式的兴起，流式检测肺癌免疫微环境成为重要的手段，但多为检测细胞亚群占比或主要细胞亚群功能，无法同时对两者进行检测。因此本研究建立了一种可靠的21色流式方案，以检测人非小细胞肺癌（non-small cell lung cancer, NSCLC）肿瘤组织中免疫细胞各亚群。 **方法** 选用细胞膜表面抗体细胞分化簇（cluster of differentiation, CD）45、CD3、CD19、CD4、CD8、程序性死亡受体1（programmed cell death 1, PD-1）、CD39、CD103、CD25、CD127、趋化因子受体8（chemokine receptor 8, CCR8）、CD56、CD11c、人类白细胞抗原（human leukocyte antigen, HLA）-DR、CD38、CD27、CD69、CD62L、CD45RA、CCR7和核酸染料L/D制定方案。首先对各抗体进行抗体滴定实验、电压优化、减一色染色和单色染色实验，确定各实验条件及检测方案后，采用6例健康成年志愿者外周血单个核细胞（peripheral blood mononuclear cells, PBMCs）标本验证方案的可行性；检测分析6例NSCLC患者的肿瘤组织样本。**结果** 采用建立的21色流式方案检测了6例NSCLC患者的肿瘤组织样本，可分析出肺癌组织中各细胞亚群的占比及主要细胞群的免疫表型和分化情况。 **结论** 成功建立的21色流式方案适用于PBMCs和NSCLC组织样本检测，为监测肺癌中免疫微环境状态提供了一种有效的新思路。

## Abstract (en)

**Background and objective** With the rise of multicolor flow cytometry, flow cytometry has become an important means to detect the immune microenvironment of lung cancer, but most of them are used to detect the proportion of cell subsets or the function of major cell subsets, and they cannot be detected at the same time. Therefore, a reliable 21-color flow cytometry protocol was established to detect the immune cell subsets in human non-small cell lung cancer (NSCLC) tumor tissues. **Methods** Cell membrane surface antibodies cluster of differentiation (CD)45, CD3, CD19, CD4, CD8, programmed cell death 1 (PD-1), CD39, CD103, CD25, CD127, chemokine receptor 8 (CCR8), CD56, CD11c, human leukocyte antigen (HLA)-DR, CD38, CD27, CD69, CD62L, CD45RA, CCR7 and nucleic acid dye L/D were used to develop the protocol. Firstly, antibody titration experiments, voltage optimization, subtraction of one color staining and single color staining experiments were carried out for each antibody, and after the experimental conditions and detection schemes were determined, the feasibility of the scheme was verified by using peripheral blood mononuclear cells (PBMCs) specimens of six healthy adult volunteers. Tumor tissue samples from 6 NSCLC patients were tested and analyzed. **Results** The established 21-color flow cytometry protocol was used to detect the tumor tissue samples of 6 NSCLC patients, and the proportion of each cell subset in lung cancer tissue, as well as the immunophenotype and differentiation of the main cell population, were analyzed. **Conclusion** The successfully established 21-color flow cytometry protocol is suitable for the detection of PBMCs and NSCLC tissue samples, which provides an effective new idea for monitoring the immune microenvironment status in lung cancer.

肺癌是全球发病率极高的癌症之一，也是癌症相关死亡的主要原因，每年约2万新发病例和1.76万死亡病例^[1]^。肺癌主要分为小细胞肺癌（small cell lung cancer, SCLC）和非小细胞肺癌（non-small cell lung cancer, NSCLC），其中NSCLC约占85%^[2]^。NSCLC由于早期症状不明显，多数发现时已属中晚期状态，且易对化疗和放疗抵抗，致5年生存率仅为10%-20%^[3]^。而肺癌的发生和进展与免疫微环境的改变相关，因此通过检测肺癌组织中免疫细胞亚群的比例可监测疾病的进展及预后等^[4,5]^。

目前，流式细胞术能快速进行各种免疫细胞量和功能的检测^[6]^，对临床诊断与治疗具有指导意义^[7]^。常规流式方案分析的参数少，需要多管联合分析，细胞需求量大，耗时较长，结果不够稳定及准确，解析各细胞亚群之间和细胞表型相互关系的能力弱^[8]^。随着流式细胞仪的更新换代，检测的荧光通道增加，多参数流式细胞术的优势逐渐显现出来^[9]^。多色流式能快速准确并稳定地检测多个指标，从而识别出罕见细胞群体，为疾病的诊断、药物开发提供强大的工具^[10]^。因此，本实验建立了在单管中加入20种荧光抗体和1种荧光染料以检测人肺癌组织中免疫细胞主要亚群的流式检测方案，可实现单管同时分析人肺癌组织中多种免疫细胞的表达和细胞亚群间的关联，并同时呈现免疫细胞功能与分化状态。现报道如下。

## 1 资料与方法

### 1.1 材料

#### 1.1.1 实验材料

6份健康志愿者外周血样本和6例肺癌样本取自四川大学华西医院，肺癌患者病理诊断均为NSCLC。受试者均签署知情同意书。

#### 1.1.2 试剂

荧光抗体分化簇（cluster of differentiation, CD）3、CD19、CD27、CD69、CD56、CD8、CD25、CD127、趋化因子受体8（chemokine receptor 8, CCR8）、CD39、CD11c、CD62L、CCR7、CD45RA购自美国BD公司；CD45、CD4、CD38、CD103、人类白细胞抗原（human leukocyte antigen, HLA）-DR、程序性死亡受体1（programmed cell death 1, PD-1）购自Biolegend公司；LIVE/DEAD Fixable Blue Dead Cell Stain（L/D）、BV stain buffer购自Sigma-Aldrich公司；胎牛血清（FBS, 500 mL, 319801-5）购自ZETA LIFE公司；IV型胶原酶（50 U/mL, 17104-019）购自Thermo Fisher公司；脱氧核糖核酸酶I（DNase I, 20 U/mL, 10104159002）购自Roche公司。

#### 1.1.3 仪器

Symphony A5流式细胞仪（美国BD Bioscience）配置355、405、488、561、640 nm激光器。

### 1.2 方法

#### 1.2.1 抗体或荧光素搭配

根据流式细胞仪的配置，包括激光和检测滤片等以及多色流式中抗原抗体结合的强弱搭配原则，即强表达抗原选择弱荧光素抗体，反之。通过多次预实验找到抗体与荧光素的最佳搭配方案。表1为所需抗体信息。

**表 1:** 21色流式检测人肺癌组织中免疫细胞亚群方案

Specificity	Fluorochrome	Clone	Purpose
CD127	BUV395	HIL-7R-M21	Treg
L/D	LIVE/DEAD fixable blue dead cell stain	-	Live/Dead
CCR7	BUV496	3D12	Tcm
CD27	BUV563	L128	Cell activation
CD19	BUV661	HIB19	B cells
CD25	BUV737	2A3	Treg
CCR8	BV421	433H	Treg
CD3	BV510	HIT3a	T cells
CD62L	BV605	DREG-56	Naive T
CD11c	BV650	B-ly6	Dendritic cells
CD69	BV711	FN50	Early cell activation
CD39	BV750	TU66	Exhaustion
CD8	BV786	RPA-T8	Cytotoxic T cells
CD4	FITC	OKT4	T helper cells
PD-1	PerCP-Cy5.5	EH12.2H7	Activation
CD38	PE	HIT2	Proliferation, adhesion
CD103	PE/Dazzle™ 594	HIB19	Resident CD8^+^ T
CD56	PE/CY7	B159	NK
CD45RA	APC (Ber-ACT8)	HI100	Temra
CD45	Alexa Fluor® 700	2D1	Leukocytes
HLA-DR	APC/CY7	L243	Activation

CD: cluster of differentiation; CCR8: chemokine receptor 8; PD-1: programmed cell death 1; NK: natural killer; HLA-DR: human leukocyte antigen-DR; Tregs: regulatory T cells; Tcm: central memory T cells.

#### 1.2.2 样本采集

##### 1.2.2.1 外周血样本处理

选取6名健康成年志愿者，男女各3名，年龄范围25-30岁。采集外周静脉血5 mL于含有EDTA抗凝剂的真空采血管中，颠倒混匀，转移到离心管中，以1:1比例，将外周血缓慢加入Ficoll液中，4 ^o^C、800 g离心20 min，吸取白膜层于5 mL RPMI-1640中，4 ^o^C、500 g离心7 min，弃上清。PBS清洗，4 ^o^C、500 g离心5 min，获取外周血单个核细胞（peripheral blood mononuclear cells, PBMCs）。将细胞重悬于PBS，计数后备用^[11]^。

##### 1.2.2.2 肺癌组织样本处理

收集NSCLC样本，共6例。手术切除的NSCLC样本，尽量去除坏死组织，PBS冲洗，置于10% FBS+RPMI-1640中，冰上运输。提前配置10 mL 0.5 mg/mL IV型胶原酶+RPMI-1640和10 mL 2% FBS+RPMI-1640，冰上预冷，待组织取回后，将组织置于2% FBS+RPMI-1640中，剪取约5 mm×5 mm×5 mm，尽量剪碎，加入5 mL消化酶，37^ o^C培养箱旋转消化30 min。消化完成后，4^ o^C、100 g离心1 min，将未消化的组织离心到管底，吸取上清。加入1倍体积的2% FBS+RPMI-1640终止消化，70 μm滤网过滤，4^ o^C、500 g离心5 min、去上清。1 mL 2% FBS+RPMI-1640重悬，40 μm滤网过滤，将细胞悬液调节成1.0×10^7^个/mL备用^[12]^。

#### 1.2.3 抗体浓度滴定

方案里的所有抗体均进行浓度滴定，共设立6个抗体的工作浓度梯度，分别为0.0、0.5、1.0、2.0、4.0及8.0 μg/mL，根据反应体系（100.00 μL）及质量浓度（200.00 μg/mL）分别加入各对应的抗体体积，即0.00、0.25、0.50、1.00、2.00和4.00 μL。以CD4抗体滴定为例，准备6支流式管，通过倍比稀释的方法，在第1管中加入96 μL PBS，其余5管中分别加入50 μL PBS。于第1管中加入4 μL CD4抗体，充分混匀后吸出50 μL至第2管，依次加入第5管，混匀后取50 μL混合液丢弃，第6管则作为空白对照组。各管中均加入50 μL已准备好的细胞悬液，完全混匀后室温避光孵育15 min，加入1 mL PBS，500 g离心5 min清洗。弃上清，300 μL PBS重悬，上机检测，收集约10^6^个Events，Flowjo 10.8.0软件分析，计算CD4的染色指数（stain index, SI）。选取最大SI值，即阳性与阴性细胞群分离度最大的值为该抗体最适浓度。其余抗体滴定及结果分析均依照此方法进行。

#### 1.2.4 电压优化、减一色对照及单色染色

在流式检测过程中，以空白对照组确定基准电压，后在此基础上依次增加40 V，共检测6个不同的电压值，再通过计算SI值找到该抗体最适电压，即SI值最大时的电压值。减一色染色指不加多色染色方案中某一种抗体或染料，验证其余抗体或染料对该相应通道的影响。单色染色为确定每个抗体或染料的最佳质量浓度后，并用于检测通道的补偿调节。

#### 1.2.5 流式方案验证

根据已确定好的流式检测条件，检测分析6例健康人PBMCs。将6份抗体及染料混合于300 μL PBS中，再分别加50 μL于各管中，分别加入已准备好的50 μL细胞悬液并充分混匀，室温避光孵育15 min，加入1 mL PBS，500 g离心5 min，清洗，弃上清，300 μL PBS重悬，上机检测，收集约10^6^个Events，Flowjo 10.8.0软件分析。

#### 1.2.6 样本检测

根据已确定好的流式检测方案，检测分析6例人NSCLC组织样本。将20种荧光抗体和L/D染料按滴定好的浓度混合于50 μL PBS中，加入已准备好的50 μL细胞悬液并充分混匀，室温下避光孵育15 min，加入1 mL PBS，400 g离心5 min，清洗，弃上清，300 μL PBS重悬，上机检测，至少收集10^6^个Events，Flowjo 10.8.0软件进行流式数据分析。

### 1.3 统计学分析

采用Flowjo 10.8.0（BD, USA）对数据进行分析。通过Flow AI软件包去除质量较差的细胞；R-tsne软件包进行降维分析；数据呈现为软件导出；呈正态分布的连续变量以均数±标准差（Mean±SD）表示。

## 2 结果

### 2.1 抗体滴定

根据SI值，该体系下CD4抗体加入的体积为0.25 μL，工作浓度为0.50 μg/mL时，SI值最大，阴性群和阳性群分离度最大；CD45、CD3、CD19、CD8、CD25、CD127、CD39、CD103、CCR8、CD38、CD69、CD27和CD56抗体加入体积均为1 μL，则工作浓度为2.00 μg/mL；CD45RA、CCR7、CD62L、HLA-DR、CD11c和PD-1抗体加入体积均为2 μL，工作浓度为4.00 μg/mL；L/D染料工作浓度为0.10 μg/mL。如图1A所示，加入0.25 μL CD4抗体时为最适工作浓度。

**图 1:**
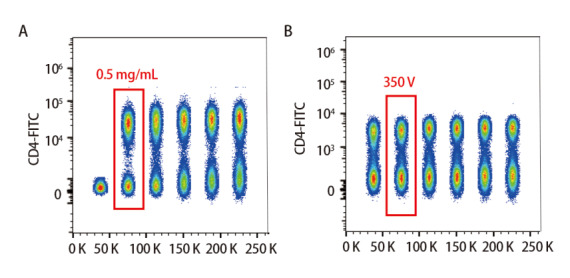
CD4抗体最适质量浓度的选择（A）和最适电压选择（B）

### 2.2 检测电压优化、补偿调节

根据得到的各个通道的SI值，确立了CD45、CD3、CD19、CD4、CD8、CD39、CD103、CD25、CD127、CCR8、CD56、CD11c、HLA-DR、CD62L、CD45RA、CCR7、CD69、CD38、CD27、PD-1抗体和L/D染料检测通道的电压为430、510、410、530、550、530、540、550、550、520、450、420、550、300、400、380、460、390、530、480和450 V。图1B为CD4抗体电压滴定结果，其余抗体均按此方法进行。补偿调节可先通过自动补偿进行调节，后再用单染样本调节并通过全染的样本进行验证。

### 2.3 样本检测与分析

已确定的流式检测方案对6名健康成年人外周血及6例人NSCLC组织进行检测，主要细胞亚群比例见表2。流式分析过程见图2，图2A为PBMCs设门步骤，图2B为肺癌组织设门步骤。分别通过Time参数设门选择信号稳定的细胞群进行分析；根据FSC-A/SSC-A设门以去除碎片和部分死细胞；FSC-A/FSCH设门，去除粘连细胞；L/D阴性为活细胞；CD45和L/D选择CD45^+^L/D^-^细胞群进行后续分析。图3为PBMCs和肺癌组织主要亚群分析策略，图3A为PBMCs分析策略，图3B为肺癌组织分析策略，分别选取CD45^+^活细胞，进行T细胞、B细胞、自然杀伤（natural killer, NK）细胞、树突状细胞（dentric cell, DC）以及B细胞亚群浆细胞（plasma cells）、组织驻留B细胞（resident memory B cells, Brm）、记忆B细胞（memory B cells）等细胞群的分析。图4来源细胞为CD4^+^/CD8^+^ T细胞的亚群进一步分析，图4A为PBMCs分析，图4B为肺癌组织分析，包括调节性T细胞（regulatory T cells, Tregs）和CD4^+^中央记忆细胞（central memory cells, Tcm）CD8^+^肿瘤浸润T细胞（tumor-infiltrating lymphocytes CD8, CD8 TILs）、PD-1分析及CD8^+^组织驻留细胞（tissue resident memory cells, Trm）的分析，以及CD4^+^初始T细胞（naive T cells, Tn）、效应记忆型T细胞（effector memory T cells, Tem）、中央记忆型T细胞（central memory T cells, Tcm）的检测。图5为t-SNE分析，选择L/D^-^和CD45^+^细胞群，分析结果。图5A为肺癌组织中除L/D和CD45外的19个抗体在肿瘤组织上的表达情况，包括T细胞、B细胞、NK细胞和Treg细胞亚群的分布情况，为整合后的6个肺癌样本。可见T细胞为CD3^+^，B细胞为CD19^+^，CD11c主要表现在非淋巴细胞；CD4和CD8高表达在T细胞；CD25、CD127和CD103主要在T细胞表达；CD38只在B细胞表达；CD27、CD39、HLA-DR、CD45RA、CCR7、CD62L和CD69在多个细胞群有表达。其中，CD25^+^CD127^-^则为Treg细胞；CD19^+^CD38^+^CD27^+^为浆细胞；CD11c^+^HLA-DR^+^为DC细胞；CD8^+^CD39^+^CD103^+^为CD8 TILs细胞；CD8^+^CD69^+^CD103^+^为Trm细胞。

**表 2:** 外周血PBMCs和NSCLC组织免疫细胞主要细胞亚群占比

Subset in leukocytes (CD45^+^)	PBMCs (Mean±SD, n=6)	NSCLC (Mean±SD, n=6)
T cells (CD3^+^)	64.67%±10.43%	71.91%±16.29%
T cells (CD3^+^CD4^+^)	32.39%±12.13%	47.59%±19.61%
T cells (CD3^+^CD8^+^)	21.64%±11.02%	41.96%±5.14%
NK T cells (CD3^+^CD56^+^)	3.98%±0.91%	1.90%±0.30%
Treg cells (CD3^+^CD4^+^CD25^+^CD127^-/low^)	0.81%±0.14%	11.15%±2.25%
CD4 T naive cells (CD3^+^CD4^+^CCR7^+^CD45RA^+^)	7.60%±1.62%	0.65%±0.28%
CD8 TILs (CD3^+^CD8^+^CD39^+^CD103^+^)	0.19%±0.03%	22.66%±2.04%
CD8 T naive cells (CD3^+^CD8^+^CCR7^+^CD45RA^+^)	10.60%±2.88%	0.14%±0.05%
B cells (CD19^+^)	9.33%±2.89%	10.38%±8.52%
Plasma cells (CD19^+^CD27^high^CD38^high^)	0.23%±0.09%	8.81%±1.56%
Brm cells (CD19^+^CD69^+^CD38^+^)	0.00%±0.00%	0.38%±0.11%
Memory B cells (CD19^+^CD27^+^CD38^-^)	0.45%±0.10%	5.98%±0.52%
NK cells (CD3^-^CD19^+^CD56^+^)	11.35%±2.90%	1.88%±0.17%
DC (CD3^-^CD19^-^CD11c^+^HLA-DR^+^)	10.33%±1.43%	39.41%±4.19%

Brm: resident memory B cells; PBMCs: peripheral blood mononuclear cells; NSCLC: non-small cell lung cancer; DC: dentric cell; TILs: tumor-infiltrating lymphocytes.

**图 2:**
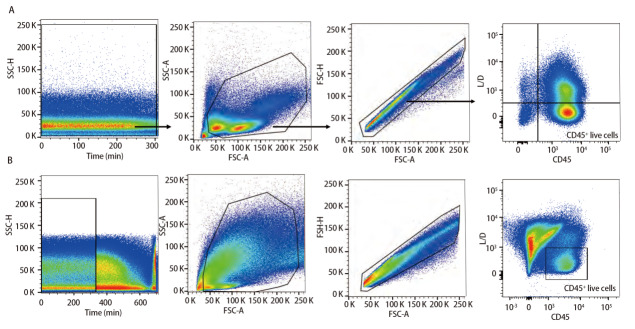
PBMCs和肺癌组织设门步骤。 A：PBMCs设门步骤；B：肺癌组织设门步骤。

**图 3:**
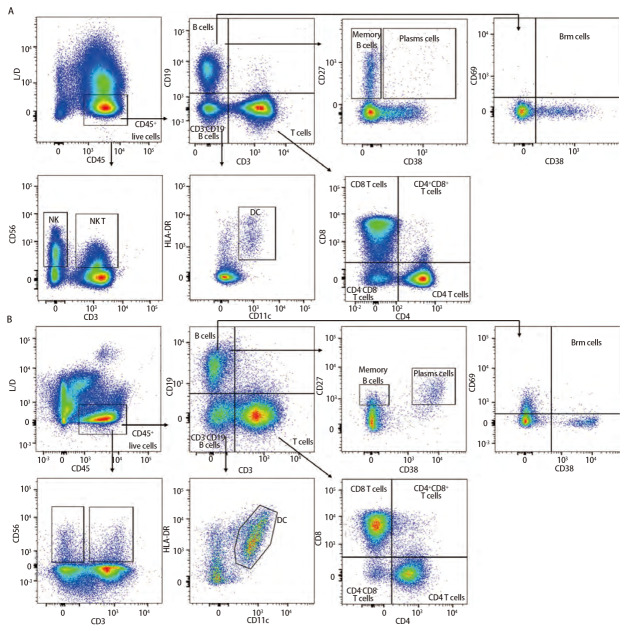
PBMCs和肺癌组织主要亚群分析策略。 A：PBMCs分析策略；B：肺癌组织分析策略。图3A、3B分别分析了PBMCs和肺癌组织的T细胞、B细胞、NK细胞、NK T细胞、DC、浆细胞等细胞亚群，结果显示不同组织之间具有差异性。

**图 4:**
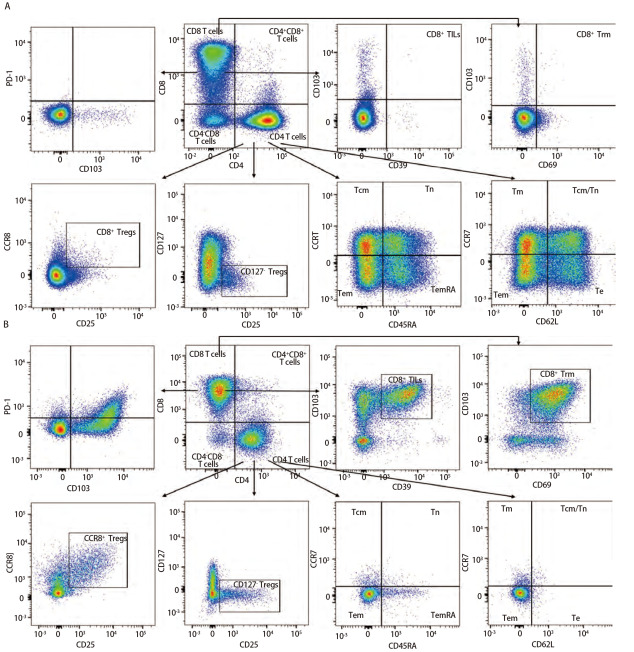
PBMCs和肺癌组织中CD4^+^/CD8^+^ T细胞亚群功能、活化分析。 A：PBMCs分析；B：肺癌组织分析。图4A、4B分别分析了PBMCs和肺癌组织的功能和活化的细胞亚群，如Tregs、CD8^+^ TILs、CD8^+^ Trm、Tcm、Tn、Tem以及PD-1的表达等，结果不同组织之间具有差异性。

**图 5:**
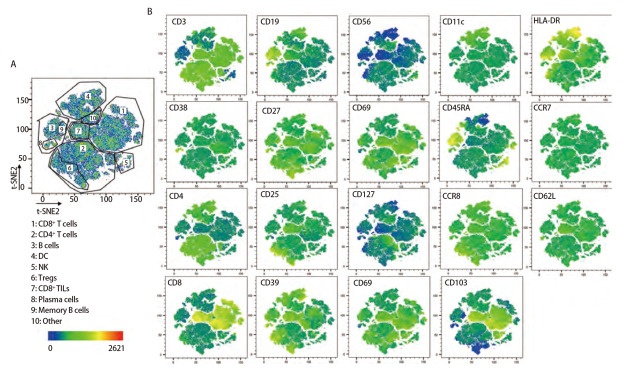
肺癌组织t-SNE分析。 呈现了6例肺癌组织的整体分布，可将其分为多各细胞亚群，包括CD8^+ ^T细胞、CD4^+ ^T细胞、NK细胞、DC等细胞亚群，图5B展现了除CD45和L/D外的其余抗体标记在整体的分布情况。

## 3 讨论

多色流式细胞术具有便捷、准确、易于标准化的特点，其因能高通量分析细胞水平而被临床和科研广泛应用。多色流式细胞术可使用多个标志物进行单细胞研究，并通过多个分析参数解析细胞，最终获得更准确界定的细胞群和更丰富的生物学信息，且具有样本使用量少、成本较低等优势^[13,14]^。例如，Keyel等^[15]^通过多色流式来鉴定混合群体（肿瘤、骨髓或血液）中的神经母细胞瘤细胞，操作简单、准确、快速。宋洋子等^[16]^通过十色流式检测外周血中T细胞亚群及活化状态，方案简单可靠，研究证实了多色流式的可行性，但并未加入外周血中其他亚群的分析。杨敏等^[17]^和Kathryn等^[18]^都曾设计了多色流式方案对肿瘤微环境中的T细胞、巨噬细胞、NK细胞等进行更细致的分型，从而获得影响肿瘤细胞进展相关的研究手段，却未纳入细胞分化等指标。

本实验建立了一个21色流式方案，以检测人NSCLC组织样本的免疫细胞亚群。Treg细胞表达CD45、CD3、CD4、CD25、FoxP3、CCR8，低或不表达CD127，其中FoxP3为核转录因子，染色前需进行细胞固定破膜，操作复杂且易损失细胞，CD127为表面标记抗原，因此本方案选择CD25^+^CD127^-/low^来定义Treg细胞^[19]^。有研究^[20]^表明CCR8是一种肿瘤浸润Treg细胞，且高表达的CCR8与多种癌症相关。在胸腺、脾脏和外周血的Treg细胞中表达量较低，本实验加入CCR8来验证外周血Treg是否浸润。CD45RA、CCR7和CD62L为细胞分化的标志，可表示细胞的分化情况。CD39和CD103作为细胞功能的标志，有报道^[21,22]^称，CD39^+^CD103^+^为肿瘤特异性及耗竭性T细胞并可识别实体瘤中的肿瘤反应性CD8^+ ^T细胞，而针对PD-1的治疗策略已被开发为包括NSCLC在内的各种癌症类型的肿瘤进展的免疫疗法^[23]^。

通过对抗体浓度和电压的优化以及多色方案的改进，本研究建立了稳定可靠的可于单管中分析人PBMCs和肺癌组织中主要细胞亚群占比的方法，PBMCs中各主要细胞亚群的比例的均值与文献^[24]^报道接近。而肺癌组织中具有个体差异性，表2为本实验所用肿瘤样本比例统计，且能清晰地看到T细胞、B细胞、NK细胞及DC等主要细胞亚群的分布，同时充分展现了这些细胞亚群之间的关联。实验操作简单，耗时较短，一定程度上节约了时间和费用。另外还可进行方案的优化或根据条件补充方案，例如将DC细胞亚群细分，如传统DCs（conventional DCs, cDCs）、血浆DCs（plasmacytoid DCs, pDCs）、朗格汉斯细胞（Langerhans cells, LCs）等纳入分析，以及其他肿瘤组织适用性验证。

综上，本实验建立了稳定的检测人NSCLC组织样本免疫细胞亚群的21色流式方案，方案适用于PBMCs与肺癌组织样本，可系统性地检测PBMCs与肺癌组织中细胞亚群含量以及亚群的功能与活化情况，细胞分群清晰，且能清楚地展现PBMCs与肺癌样本中细胞分群的差异性，为流式监测疾病的进展等提供了一种可靠的新方案。
